# Analysis on time delay of tuberculosis among adolescents and young adults in Eastern China

**DOI:** 10.3389/fpubh.2024.1376404

**Published:** 2024-04-08

**Authors:** Rui Ge, Guoying Zhu, Min Tian, Zhigang Hou, Weizhe Pan, Hao Feng, Kui Liu, Qinfeng Xiao, Zhongwen Chen

**Affiliations:** ^1^Jiaxing Center for Disease Control and Prevention, Jiaxing, Zhejiang, China; ^2^Nanhu Center for Disease Control and Prevention, Jiaxing, Zhejiang, China; ^3^Department of Tuberculosis Control and Prevention, Zhejiang Provincial Center for Disease Control and Prevention, Hangzhou, Zhejiang, China; ^4^The First Hospital of Jiaxing, Jiaxing, Zhejiang, China

**Keywords:** tuberculosis, delay, patient delay, health system delay, influencing factors, logistic regression

## Abstract

**Background:**

Tuberculosis (TB) is recognized as a significant global public health concern. Still, there remains a dearth of comprehensive evaluation regarding the specific indicators and their influencing factors of delay for adolescents and young adults.

**Methods:**

All notified pulmonary TB (PTB) patients in Jiaxing City were collected between 2005 and 2022 from China’s TB Information Management System. Logistic regression models were conducted to ascertain the factors that influenced patient and health system delays for PTB cases, respectively. Furthermore, the impact of the COVID-19 pandemic on local delays has been explored.

**Results:**

From January 1, 2005 to December 31, 2022, a total of 5,282 PTB cases were notified in Jiaxing City, including 1,678 adolescents and 3,604 young adults. For patient delay, female (AOR: 1.18, 95%CI: 1.05–1.32), PTB complicated with extra-pulmonary TB (AOR: 1.70, 95% CI: 1.28–2.26), passive case finding (AOR: 1.46, 95% CI: 1.07–1.98) and retreatment (AOR: 1.52, 95% CI: 1.11–2.09) showed a higher risk of delay. For health system delay, minorities (AOR: 0.69, 95% CI: 0.53–0.90) and non-students (AOR: 0.83, 95% CI: 0.71–0.98) experienced a lower delay. Referral (AOR: 1.46, 95% CI: 1.29–1.65) had a higher health system delay compared with clinical consultation. Furthermore, county hospitals (AOR: 1.47, 95% CI: 1.32–1.65) and etiological positive results (AOR: 1.46, 95% CI: 1.30–1.63) were associated with comparatively high odds of patient delay. Contrarily, county hospitals (AOR: 0.88, 95% CI: 0.78–1.00) and etiological positive results (AOR: 0.67, 95% CI: 0.59–0.74) experienced a lower health system delay. Besides, the median of patient delay, health system delay, and total delay during the COVID-19 pandemic were significantly lower than that before.

**Conclusion:**

In general, there has been a noteworthy decline in the notification rate of PTB among adolescents and young adults in Jiaxing City while the declining trend was not obvious in patient delay, health system delay, and total delay, respectively. It also found factors such as gender, case-finding method, and the hospital level might influence the times of seeking health care and diagnosis in health agencies. These findings will provide valuable insights for refining preventive and treatment strategies for TB among adolescents and young adults.

## 1 Introduction

Tuberculosis is a chronic respiratory infectious disease caused by *Mycobacterium tuberculosis* (Mtb), which seriously endangers human health (1). Prior to the COVID-19 pandemic, TB held the unfortunate distinction of being the primary cause of death caused by a single infectious agent, surpassing even HIV/AIDS in terms of mortality rates (2). An estimated 2.28 million adolescents and young adults were diagnosed with TB worldwide in 2022, which accounted for approximately 21.5% of all newly reported TB patients (2). Adolescents and young adults are particularly susceptible to TB, as this age group commonly experiences a significant increase in both exposure to TB and rising pressure derived from learning and work (3). In the high burden countries, adolescents and young adults make up a substantial proportion of the general population and a substantial proportion of TB patients. The incidence of TB in China ranks third around the world, and it is also listed as one of the 22 high-burden countries (2). According to the release of the Global TB Report in 2023, there were an estimated 748,000 TB patients and 30,000 died from the disease in 2022 in China, while the reported rate of TB among young and older people was higher than other subgroups (2, 4). Thus, controlling TB among adolescents and young adults is of paramount importance for effectively managing the TB epidemic in China.

Although the strategy of passive case-finding was the mainstream of prevention and control of TB in most countries, it could not hold back TB epidemic and realize the goal of ending TB (5). To ensure effective control of TB and minimize its impact, it is crucial to diagnose the disease early and prompt sufficient treatment. By promptly identifying TB in individuals, treatment could be initiated without delay and prevent further progression of the TB within their body (6). Moreover, timely treatment initiation also plays a significant role in reducing the risk of community transmission. Various factors contribute to the delay (7–9). Consequently, it is imperative to identify them to implement interventions that will optimize the effectiveness of TB control programs.

This study aimed to analyze the changed trend of delay and its influencing factors of TB among adolescents and young adults in Jiaxing City, which would provide a basis for formulating corresponding prevention and control measures, to promote early detection and treatment of TB.

## 2 Materials and methods

### 2.1 Geographic information

**Figure 1:**
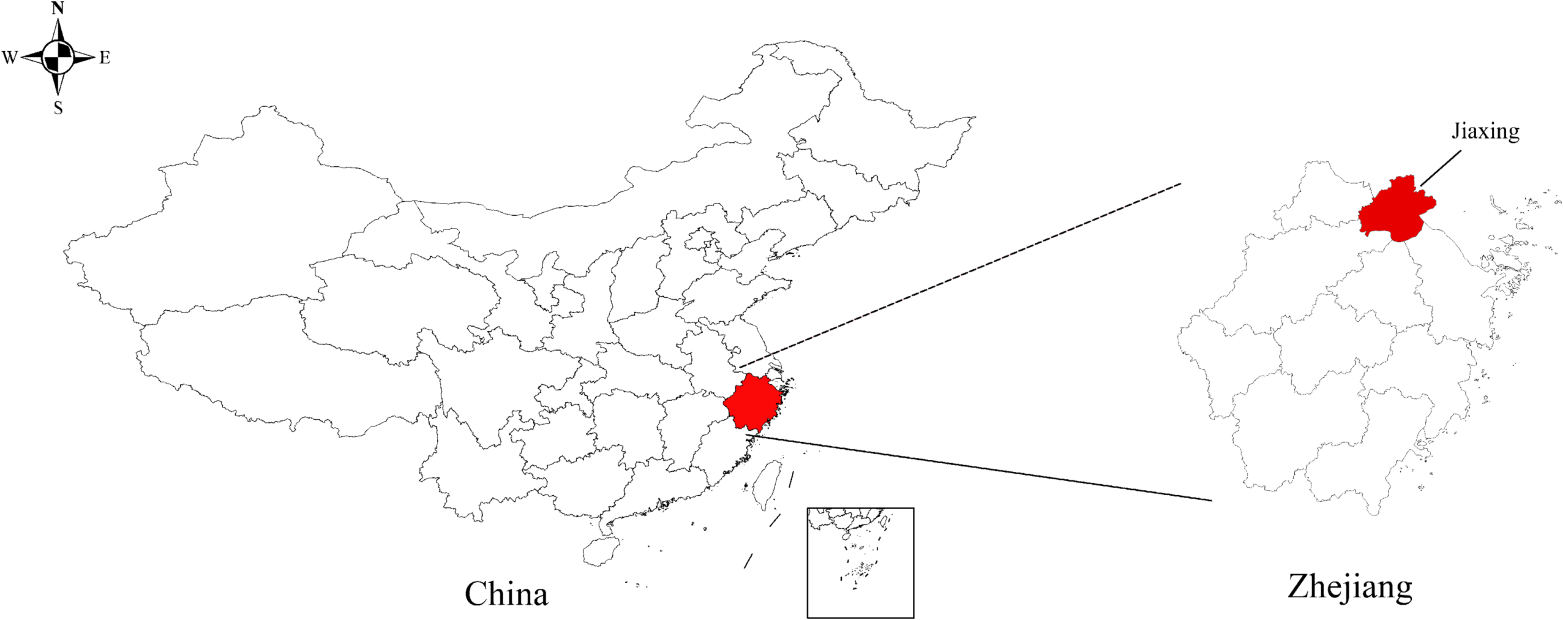
The geographical location of Jiaxing City.

Jiaxing City, one of the 11 cities under the jurisdiction of Zhejiang Province, is located in the southeast coastal area of China. It consists of 7 counties like Nanhu, Xiuzhou, Haining, Tongxiang, Jiashan, Pinghu, and Haiyan. The total area of Jiaxing City is 3,915 km^2^. According to the seventh population census, the permanent population in Jiaxing City was 5,400,868, with nearly 722,920 people aged 10–24 years, accounting for 13.39% of the total population. Additionally, Jiaxing City has an average annual temperature of 15.9°C and an annual cumulative precipitation of 1168.6 mm. The location of Jiaxing City is shown in Figure 1.

### 2.2 Participants

In this study, we collected all notified pulmonary TB (PTB) cases aged 10–24 years between 2005 and 2022 from China’s TB information management system, which included the details of demographic, treatment, and laboratory testing information of patients (10).

### 2.3 Definition

All included pulmonary PTB patients were diagnosed according to the National Diagnostic Criteria for Pulmonary Tuberculosis (WS288-2008 and WS288-2017) and the Classification of Tuberculosis (WS196-2001 and WS196-2017) in China, including laboratory-confirmed TB and clinically diagnosed TB (11, 12). Laboratory-confirmed PTB was defined as people who were diagnosed with bacteriological evidence from a sputum smear, sputum culture, or suitable rapid diagnostic technology such as Gene-Xpert. These tests are instrumental in identifying and confirming cases of PTB through the detection of specific pathogens. Additionally, clinically diagnosed PTB refers to the comprehensive diagnosis based on the patient’s epidemiological history, clinical symptoms, imaging manifestations, and negative sputum smear results (13).

Patient delay was defined as the interval days between the first appearance of PTB symptoms and the first visit to a medical institution. Health system delay refers to the interval days between the first visit to a medical institution and the confirmed diagnosis of PTB (14). Total delay consisted of patient delay and health system delay (15). The distribution of different delays at various time points did not follow a normal pattern, therefore median days were utilized as a threshold to define delays. Case-finding methods were divided into active and passive methods: (1) Active method included active screening and physical examination; (2) Passive method included directly seeking medical care and recommendations from other health institutions, etc.

### 2.4 Statistical analysis

Descriptive analysis was conducted to present the general epidemiological characteristics of the demographic information. Continuous variables were reported using the median and interquartile range (IQR), while categorical variables were presented as counts and proportions. The Mann–Whitney test was utilized to compare two groups, while the Kruskal-Wallis test was employed for comparing three or more groups. The influencing factors of delay were analyzed using univariate and multivariate logistic regression models. Additionally, the annual percentage change (APC) of TB incidence among adolescents and young adults from 2005 to 2022 was calculated by Joinpoint 5.0.2. The geographic information of Jiaxing City was visualized using ArcGIS 10.9. SPSS 26.0 was used for statistical analysis. All variables significantly associated with the delay were identified based on a *p*-value less than 0.05.

## 3 Results and discussion

### 3.1 General epidemiological characteristics

From January 1, 2005 to December 31, 2022, a total of 5,282 PTB cases were notified in Jiaxing City, including 1,678 adolescents and 3,604 young adults. This accounts for approximately 15.32% (5,282/34487) of the total number of PTB cases during that period. The gender ratio was 1.61:1, with males slightly outnumbering females. Students accounted for approximately 17% of all cases, and the majority of cases (92.3%) were of Han nationality. Additionally, migrant PTB cases made up a significant proportion, accounting for 84.17% of the total cases. The notification rate of this specific group showed two distinct stages of change during this period. Initially, the notification rate increased from 32.59 per 100,000 in 2005 to 59.84 per 100,000 in 2007 (APC = 37.76%, *p* < 0.05). However, the notification rate then decreased from 64.76 per 100,000 in 2008 to 12.86 per 100,000 in 2022, indicating a downtrend (APC = −7.43%, *p* < 0.05).

### 3.2 Delays of PTB among adolescents and young adults in Jiaxing

**Figure 2:**
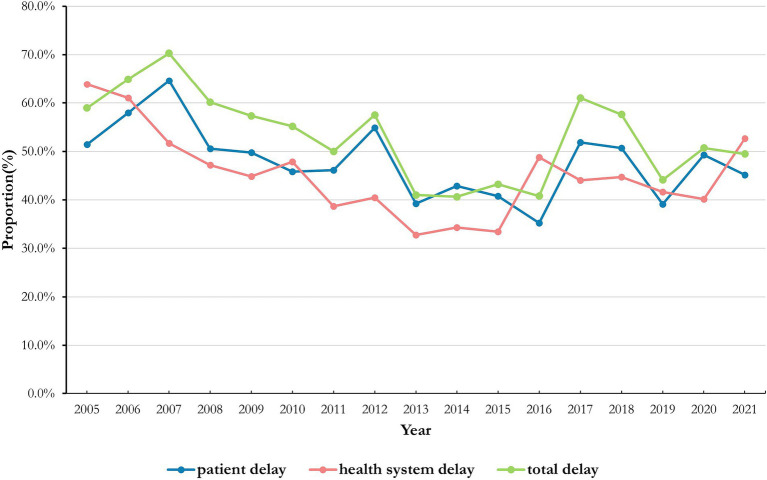
The annual proportion of PTB delays in Jiaxing City, 2005–2022.

The median of total delay was 13 (7–34) in Jiaxing City and the proportion of total delay was 54.17% (2,861/5,282). Meanwhile, the median for patient delay was 11 (3–30) and the proportion of patient delay was 48.85% (2,580/5,282). The median of health system delay was 1 (0, 4) and the proportion of health system delay was 46.19% (2,440/5,282). The annual proportion of delays is presented in Figure 2. There were no significant annual differences in patient delay, health system delay, and total delay during the study period (all *p* > 0.05). The results are shown in Table 1.

**Table 1:** Demographic characteristics of adolescents and young adults with PTB from 2005 to 2021 in Jiaxing City.

Variables	2005	2006	2007	2008	2009	2010	2011	2012	2013	2014	2015	2016	2017	2018	2019	2020	2021	2022
Total																		
Number of cases	221	346	416	441	354	424	397	362	299	339	315	287	250	218	217	161	142	93
Notification rate (per 100,000)	32.59	50.40	59.84	64.76	52.11	61.70	53.41	38.26	31.51	37.04	34.36	31.89	27.69	24.43	34.22	25.99	19.64	12.86
Gender, n (%)																		
Male	140 (63.35)	215 (62.14)	251 (60.34)	257 (58.28)	201 (56.78)	248 (58.49)	254 (63.98)	218 (60.22)	192 (64.21)	224 (66.08)	209 (66.35)	189 (65.85)	172 (68.80)	134 (61.47)	143 (65.90)	77 (47.83)	87 (61.27)	45 (48.39)
Female	81 (36.65)	131 (37.86)	165 (39.66)	184 (41.72)	153 (43.22)	176 (41.51)	143 (36.02)	144 (39.78)	107 (35.79)	115 (33.92)	106 (33.65)	98 (34.15)	78 (31.20)	84 (38.53)	74 (34.10)	84 (52.17)	55 (38.73)	48 (51.61)
Age, n (%)																		
10–19	76 (34.39)	113 (32.66)	149 (35.82)	132 (29.93)	115 (32.49)	137 (32.31)	107 (26.95)	113 (31.22)	98 (32.78)	112 (33.04)	103 (32.70)	97 (33.80)	77 (30.80)	74 (33.94)	66 (30.41)	39 (24.22)	39 (27.46)	31 (33.33)
20–24	145 (65.61)	233 (41.91)	267 (34.86)	309 (32.88)	239 (40.96)	287 (34.20)	290 (36.52)	249 (40.06)	201 (48.49)	227 (42.77)	212 (46.03)	190 (50.52)	173 (58.00)	144 (66.51)	151 (66.82)	122 (90.06)	103 (102.11)	62 (155.91)
Ethnics, n (%)																		
Han	221 (100.00)	346 (100.00)	416 (100.00)	440 (99.77)	334 (94.35)	407 (95.99)	373 (93.95)	353 (97.51)	284 (94.98)	318 (93.81)	291 (92.38)	248 (86.41)	228 (91.20)	197 (90.37)	204 (94.01)	152 (94.41)	137 (96.48)	82 (88.17)
Minorities	0 (0.00)	0 (0.00)	0 (0.00)	1 (0.23)	20 (5.65)	17 (4.01)	24 (6.05)	9 (2.49)	15 (5.02)	21 (6.19)	24 (7.62)	39 (13.59)	22 (8.80)	21 (9.63)	13 (5.99)	9 (5.59)	5 (3.52)	11 (11.83)
Occupation, n (%)																		
Student	27 (12.22)	45 (13.01)	49 (11.78)	63 (14.29)	63 (17.80)	53 (12.50)	42 (10.58)	47 (12.98)	36 (12.04)	52 (15.34)	33 (10.48)	50 (17.42)	30 (12.00)	38 (17.43)	50 (23.04)	36 (22.36)	38 (26.76)	27 (29.03)
Others	194 (87.78)	301 (86.99)	367 (88.22)	378 (85.71)	291 (82.20)	371 (87.50)	355 (89.42)	315 (87.02)	263 (87.96)	287 (84.66)	282 (89.52)	237 (82.58)	220 (88.00)	180 (82.57)	167 (76.96)	125 (77.64)	104 (73.24)	66 (70.97)
Residence, n (%)																		
Local	99 (44.80)	103 (29.77)	110 (26.44)	78 (17.69)	37 (10.45)	3 (0.71)	3 (0.76)	7 (1.93)	14 (4.68)	71 (20.94)	65 (20.63)	55 (19.16)	56 (22.40)	24 (11.01)	34 (15.67)	30 (18.63)	25 (17.61)	22 (23.66)
Migrant	122 (55.20)	243 (70.23)	306 (73.56)	363 (82.31)	317 (89.55)	421 (99.29)	394 (99.24)	355 (98.07)	285 (95.32)	268 (79.06)	250 (79.37)	232 (80.84)	194 (77.60)	194 (88.99)	183 (84.33)	131 (81.37)	117 (82.39)	71 (76.34)
Types of delay, (Median, IQR)																		
Patient delay	9 (3, 31)	12.5 (3, 33)	16.5 (5, 55)	23 (6, 54)	12 (3, 34)	11 (4, 30)	9 (4, 30)	10 (4, 21)	13 (5, 24)	9 (5 16)	9 (3, 24)	7 (0, 29)	5 (1, 16)	13 (4, 30)	12 (5, 27)	8 (3, 17)	11 (4, 26)	9 (3–29)
Health system delay	2 (1, 5)	2 (1, 6)	2 (1, 5)	2 (1, 4)	1 (0, 4)	1 (0, 4)	1 (0, 4)	1 (0, 3)	1 (0, 3)	1 (0, 2)	1 (0, 2)	0 (0, 2)	1 (0, 4)	1 (0, 4)	1 (0, 4)	0 (0, 4)	0 (0, 4)	2 (0, 6)
Total delay	13 (6, 37)	19 (7, 50)	28 (9, 68)	31 (10, 66)	19 (7, 41)	16 (7, 32)	16 (6, 33)	13.5 (7, 25)	15 (9, 29)	11 (7, 20)	11 (5, 28)	10 (0, 31)	9.5 (3, 23)	16 (8, 33)	17 (7, 32)	11 (6, 22)	14 (7, 29)	13 (7, 29)

### 3.3 Influencing factors analysis of delay

The univariate and multiple logistic models were conducted to estimate the influencing factors of patient delay and health system delay. For patient delay, female (AOR: 1.18, 95%CI: 1.05–1.32), PTB complicated with extra-pulmonary TB (AOR: 1.70, 95% CI: 1.28–2.26), passive case-finding (AOR: 1.46, 95% CI: 1.07–1.98) and retreatment (AOR: 1.52, 95% CI: 1.11–2.09) showed a higher delay. For health system delay, minorities (AOR: 0.69, 95% CI: 0.53–0.90) and non-students (AOR: 0.83, 95% CI: 0.71–0.98) experienced a lower delay. Referral (AOR: 1.46, 95% CI: 1.29–1.65) had a higher health system delay compared with clinical consultation.

Furthermore, county hospitals (AOR: 1.47, 95% CI: 1.32–1.65) and etiological positive (AOR: 1.46, 95% CI: 1.30–1.63) were associated with high odds of patient delay. Contrarily, county hospitals (AOR:0.88, 95% CI: 0.78–1.00) and etiological positive (AOR: 0.67, 95% CI: 0.59–0.74) experienced a lower health system delay. This information is presented in Tables 2, 3.

**Table 2:** The influencing factors analysis of patient delay among adolescents and young adults with PTB.

Variables	No. of cases	Patient delay (%)	Crude OR (95% CI)	*p*-value	Adjusted OR (95% CI)	*p*-value
Gender						
Male	3,256	1,531 (47.02)	Reference		Reference	
Female	2026	1,049 (51.78)	1.21 (1.08–1.35)	0.001	**1.18 (1.05–1.32)**	**0.004**
Age						
10–19	1,678	841 (50.12)	Reference		Reference	
20–24	3,604	1739 (48.25)	0.93 (0.83–1.04)	0.206	0.93 (0.82–1.05)	0.218
Ethnics						
Han	5,031	2,445 (48.60)	Reference		Reference	
Minorities	251	135 (53.78)	1.23 (0.96–1.59)	0.109	1.02 (0.78–1.32)	0.900
Occupation						
Student	779	373 (47.88)	Reference		Reference	
Others	4,503	2,207 (49.01)	1.05 (0.90–1.22)	0.560	1.03 (0.87–1.21)	0.749
Residence						
Local	836	409 (48.92)	Reference		Reference	
Migrant	4,446	2,171 (48.83)	1.00 (0.86–1.16)	0.961	1.00 (0.86–1.17)	0.982
Case-finding methods						
Active	182	77 (42.31)	Reference		Reference	
Passive	5,100	2,503 (49.08)	1.31 (0.97–1.77)	0.073	**1.46 (1.07–1.98)**	**0.016**
Classification						
PTB solo	5,067	2,451 (48.37)	Reference		Reference	
PTB complicated with extra-pulmonary TB	215	129 (60.00)	1.60 (1.21–2.12)	0.001	**1.70 (1.28–2.26)**	**0.000**
Season of onset						
First quarter	1,321	676 (51.17)	Reference		Reference	
Second quarter	1,488	709 (47.65)	0.87 (0.75–1.01)	0.062	0.87 (0.75–1.01)	0.064
Third quarter	1,359	668 (49.15)	0.92 (0.79–1.07)	0.296	0.90 (0.78–1.05)	0.198
Fourth quarter	1,114	527 (47.31)	0.86 (0.73–1.01)	0.057	0.87 (0.74–1.02)	0.084
Hospital level of first diagnosis						
Provincial and municipal hospitals	2,304	1,001 (43.45)	Reference		Reference	
County hospitals	2,978	1,579 (53.02)	1.47 (1.32–1.64)	0.000	**1.47 (1.32–1.65)**	**0.000**
Treatment history						
New	5,113	2,480 (48.50)	Reference		Reference	
Retreatment	169	100 (59.17)	1.54 (1.13–2.10)	0.007	**1.52 (1.11–2.09)**	**0.009**
Etiological diagnosis						
Negative	2,877	1,285 (44.66)	Reference		Reference	
Positive	2,303	1,243 (53.97)	1.45 (1.30–1.62)	0.000	**1.46 (1.30–1.63)**	**0.000**
Unknown	102	52 (50.98)	1.29 (0.87–1.91)	0.209	1.29 (0.86–1.93)	0.213

The bold values mean that statistical significance.

**Table 3:** The influencing factors analysis of health system delay among adolescents and young adults with TB.

Variables	No. of cases	Health system delay (%)	Crude OR (95% CI)	*p*-value	Adjusted OR (95% CI)	*p*-value
Gender						
Male	3,256	1,511 (46.41)	Reference		Reference	
Female	2,026	929 (45.85)	0.98 (0.88–1.09)	0.695	0.99 (0.89–1.11)	0.899
Age						
10–19	1,678	785 (46.78)	Reference		Reference	
20–24	3,604	1,655 (45.92)	0.97 (0.86–1.09)	0.559	1.00 (0.88–1.13)	0.955
Ethnics						
Han	5,031	2,342 (46.55)	Reference		Reference	
Minorities	251	98 (39.04)	0.74 (0.57–0.95)	0.020	**0.69 (0.53–0.90)**	**0.006**
Occupation						
Student	779	389 (49.94)	Reference		Reference	
Others	4,503	2,051 (45.55)	0.84 (0.72–0.98)	0.023	**0.83 (0.71–0.98)**	**0.032**
Residence						
Local	836	384 (45.93)	Reference		Reference	
Migrant	4,446	2,056 (46.24)	1.01 (0.87–1.17)	0.869	0.98 (0.84–1.15)	0.818
Sources of patients						
Clinical consultation	2,841	1,230 (43.29)	Reference		Reference	
Referral	2,282	1,141 (50.00)	1.31 (1.17–1.46)	0.000	**1.46 (1.29–1.65)**	**0.000**
others	159	69 (43.40)	1.00 (0.73–1.39)	0.980	1.06 (0.76–1.47)	0.748
Classification						
PTB solo	5,067	2,341 (46.20)	Reference		Reference	
PTB complicated with extra-pulmonary TB	215	99 (46.05)	0.99 (0.76–1.31)	0.965	0.91 (0.69–1.20)	0.502
Season of onset						
First quarter	1,321	623 (47.16)	Reference		Reference	
Second quarter	1,488	676 (45.43)	0.93 (0.80–1.08)	0.358	0.92 (0.79–1.07)	0.271
Third quarter	1,359	622 (45.77)	0.95 (0.81–1.10)	0.470	0.95 (0.82–1.11)	0.518
Fourth quarter	1,114	519 (46.59)	0.98 (0.83–1.15)	0.778	0.97 (0.83–1.14)	0.722
Hospital level of first diagnosis						
Provincial and municipal hospitals	2,304	1,070 (46.44)	Reference		Reference	
County hospitals	2,978	1,370 (46.00)	0.98 (0.88–1.10)	0.752	**0.88 (0.78–1.00)**	**0.049**
Treatment history						
New	5,113	2,365 (46.25)	Reference		Reference	
Retreatment	169	75 (44.38)	0.93 (0.68–1.26)	0.630	1.05 (0.77–1.44)	0.741
Etiological diagnosis						
Negative	2,877	1,449 (50.36)	Reference		Reference	
Positive	2,303	940 (40.82)	0.68 (0.61–0.76)	0.000	**0.67 (0.59–0.74)**	**0.000**
Unknown	102	51 (50.00)	0.99 (0.66–1.46)	0.942	0.97 (0.65–1.44)	0.880

The bold values mean that statistical significance.

### 3.4 Impact of COVID-19

Considering the impact of the COVID-19 pandemic, we classified the cases notified around 2020 and compared the differences in delays using the Mann–Whitney test. The median of patient delay, health system delay, and total delay of 2020–2022 were significantly lower than those of 2005–2019, respectively. This information is presented in Table 4.

**Table 4:** Comparison of delays between 2005–2019 and 2020–2022.

Types of delay (median, IQR)	2005–2019 (*n* = 4,886)	2020–2022 (*n* = 396)	*p*-value
Patient delay	11 (3, 31)	9 (3, 23)	0.038
Health system delay	1 (0, 4)	0 (0, 4)	0.002
Total delay	15 (7, 35)	13 (6, 26)	0.024

## 4 Discussion

In this study, we conducted a comprehensive analysis of the PTB notification rate among adolescents and young adults in Jiaxing City over the last dozen years, as well as patient delay and health system delay. From 2005 to 2022, a total of 5,282 PTB patients aged 10–24 years were notified in Jiaxing City, accounting for 15.32% of the overall cases. This percentage aligns with the global estimates (2). In recent years, China has implemented the directly observed treatment short-course (DOTS) and the “End TB strategy” to enhance the prevention and control of tuberculosis (16–18). As a result of these strategies, the incidence rate and number of notified cases among individuals aged 10–24 years in Jiaxing City have shown a declining trend since 2008. The median patient delay and health system delay in Jiaxing were 11 and 1, respectively, which were lower than the overall levels observed in China and some findings from other countries (19–22). Despite the median time of PTB delay in Jiaxing City being consistently low, there was no significant decrease in the risk of delay between 2005 and 2022. Given seeking medical care with delay exacerbating the burden of tuberculosis and escalating the financial strain on patients, families, and the wider public health system, it therefore highlighted the ongoing need for improved control measures to reduce delays across various stages (23). Besides, a series of specific interventions such as targeted educational campaigns, community outreach programs, the improvement of healthcare infrastructure, and innovative approaches to case detection and treatment initiation also should be strengthened in the future.

In addition, we performed a thorough analysis of various factors, such as gender, age, ethnicity, occupation, and the mode of case-finding, to examine the relationship of influencing factors and delay. Our findings indicated that there was a higher proportion of patient delay among females than males. That is to say, females tend to delay the behavior of seeking health care when presenting PTB symptoms (24). This result was consistent with some available study that listed females emerged as a significant factor associated with a reduced likelihood of seeking diagnosis and treatment for TB (25). In general, airborne pathogens are expected to pose an equal biological risk to all individuals. The reasons for the discrepancy of delay among gender might be attributed to the susceptibility of stigma and vulnerability to PTB. Females were found to have a higher level of TB-related stigma compared to males, which can limit their access to medical care (26). Additionally, due to their vulnerability and unique physiological characteristics, female patients may present milder symptoms than their male counterparts, potentially leading to delays in seeking medical care (27). Thus, the stigma caused by TB needs to be eliminated through extensive and effective health education and health promotion. PTB patients with extra-pulmonary TB demonstrated a higher likelihood of experiencing patient delay. Specifically, patients with extra-pulmonary TB were found to be 1.70 times more likely to delay the diagnosis compared to PTB (AOR = 1.70, 95%CI: 1.28–2.26). Tuberculosis, as a disease associated with poverty, is linked to the timeliness of disease detection, severity, and prognosis with the economic status of patients (28). It is speculated that PTB patients with extra-pulmonary TB may be constrained by their economic conditions and understanding of the disease, thereby increasing the risk of patient delay. Furthermore, the presence of PTB along with extra-pulmonary TB could also lead to the complex clinical presentations and make the PTB diagnosis difficult (29). These findings align with similar studies indicating that PTB patients with extra-pulmonary TB are more prone to delay seeking medical care compared to those with only PTB (29, 30). This emphasizes the importance of providing specific attention in clinical practice to PTB patients with extra-pulmonary TB to enhance the early detection to prevent further complications and transmission.

Retreatment was identified as an independent risk factor for patient delay in our study. Retreatment PTB refers to patients who have received inconsistent anti-TB treatment for more than 1-month, experienced treatment failure, and subsequently relapsed (31). These individuals tend to undervalue their health status and lack the motivation to actively seek medical care. This reminder highlights the importance of paying attention to retreated patients in TB surveillance and active case finding, as this is also important in preventing the emergence of drug resistance (31). Moreover, this study found that active case-finding has been demonstrated to be more effective in reducing patient delay compared to passive case-finding.

Our study also revealed several findings regarding the factors influencing health system delay. We observed that minorities and non-students had a lower risk of experiencing health system delay. Our further analysis found that 90% of minorities and non-students were from workers. In Zhejiang Province, nearly all employees were requested for the pre-employment physical examinations and annual health check-ups. Once people with suspected PTB were considered, the company will require them to seek medical care for a definitive diagnosis, thereby reducing potential health system delay among these groups. Meanwhile, student population generally had heavy study loads, and faced with potential suspension of schooling and possible stigma, leading to poor pre-diagnostic compliance and exacerbating health system delay. In this study, the risk was 1.46 times higher for referral patients compared to those who sought clinical consultation directly. This could be attributed to the fact that referral patients seek medical care across different healthcare institutions, leading to a prolonged diagnostic process. Despite the median health system delay in Jiaxing City being only 1 day, in the context of rapid diagnostic technology taking only 4 h to diagnose, there is still a need for improvement in implementing control measures aimed at reducing health system delay across various stages.

Patient delay at the county level of first diagnosis was found to be higher compared to city-level and higher medical institutions, which might be attributed to characteristics of the visiting population. Patients seeking medical care in county-level hospitals generally have limited economic conditions, milder symptoms, and lower awareness of the disease compared to those in higher-level hospitals. Nevertheless, county hospitals presented a lower health system delay than higher-level hospitals. With the development of primary healthcare services and improved TB screening capabilities in China (16, 18), the health system delay in county-level hospitals are relatively low. People with TB can receive timely TB diagnosis and treatment services when seeking medical care. Conversely, higher-level hospitals receive mainly referrals with complicated condition resulting in a longer time to diagnosis than county hospitals. Our study has indicated that patients with positive etiology are at a higher risk of patient delay and a lower health system delay. The patient delay may be related to the interactive influence and causal transformation between etiology and outcome (32). Meanwhile, with improved etiological detection in hospitals and the availability of Mtb testing, people with a positive etiology are more likely to receive confirmation.

The COVID-19 pandemic is bound to have an impact on the detection and diagnosis of tuberculosis. To assess its effect on the identification of PTB among adolescents and young adults in Jiaxing City more accurately, we compared various types of delays between two time periods: 2005–2019 and 2020–2022. An obvious decline of notified PTB cases was observed in our study during 2020–2022, which was consistent with some studies in China (33–35). The reason may be partly attributed to the implementation of COVID-19 prevention and control policies. Some implementations of non-pharmaceutical interventions in China, such as social distancing and community containment measures, effectively prevented the transmission of COVID-19 until the end of 2022 (19, 36). Interestingly, our study found a significant reduction in delays after 2019. We speculated that the increased awareness for seeking health care among young people, especially presenting serious respiratory symptom like COVID-19 may lead to the increased identification of PTB cases, thereby reducing patient delay. Additionally, the enhancement of etiological detection in hospitals also played a pivotal role in mitigating the health system delay. Still, some rigorous containment measures during the pandemic might impeded the access of non-urgent patients or asymptomatic patients to hospitals, lowering the finding of active PTB and thereby increasing the risk of TB infection in household. Besides, the afraid of the stigma associated with COVID-19 might also promote the refuse to seek medical care among some asymptomatic or mildly symptomatic patients (37, 38). Thus, TB epidemic clustered in family or some communities need be taken into account in the context of the post-pandemic era.

### 4.1 Limitations

It is important to acknowledge certain limitations of this study. Firstly, the onset time of the research subjects was self-reported by the patients, which existed the possibility of recall bias. Meanwhile, the presence of missing data also had an impact on the results of the study. Additionally, the data utilized in this study was obtained solely from the national TB information management system, without conducting further epidemiological investigations. Some variables like income level and access to healthcare services were hard to acquire, which hindered the comprehensive analysis. It is suggested that longitudinal and qualitative research should be conducted in the future. It is also important to explore the multiple reasons that contribute to delays among adolescents and young adults with PTB and to evaluate the effectiveness of interventions.

## 5 Conclusion

Over the past decades, there has been a noteworthy decline in the notification rate of PTB among adolescents and young adults in Jiaxing City while the declining trend was not obvious in patient delay, health system delay, and total delay, respectively. It also found factors such as gender, case-finding method, and the hospital level might influence the times of seeking health care and diagnosis in health agencies. These findings will provide valuable insights for refining preventive and treatment strategies for TB among adolescents and young adults.

## Data availability statement

The raw data supporting the conclusions of this article will be made available by the authors, without undue reservation.

## Ethics statement

The studies involving humans were approved by the Ethics Committee of the Jiaxing Municipal Center for Disease Control and Prevention. The studies were conducted in accordance with the local legislation and institutional requirements. Written informed consent for participation was not required from the participants or the participants’ legal guardians/next of kin in accordance with the national legislation and institutional requirements.

## Author contributions

RG: Funding acquisition, Writing – original draft, Formal analysis, Investigation. GZ: Data curation, Investigation, Writing – original draft. MT: Formal analysis, Investigation, Visualization, Writing – original draft. ZH: Data curation, Investigation, Writing – review & editing. WP: Data curation, Investigation, Writing – review & editing. HF: Investigation, Project administration, Writing – review & editing. KL: Funding acquisition, Project administration, Validation, Writing – review & editing. QX: Data curation, Software, Supervision, Writing – review & editing. ZC: Project administration, Resources, Supervision, Writing – review & editing.
